# A conservative treatment option for a single missing premolar using a partial veneered restoration with the SR Adoro system

**DOI:** 10.4103/0972-0707.66722

**Published:** 2010

**Authors:** Roheet A Khatavkar, Vivek S Hegde

**Affiliations:** Department of Conservative Dentistry and Endodontics, M.A. Rangoonwala Dental College, Pune, India

**Keywords:** Heat-cured composite, partial veneered restoration, resin-bonded prosthesis, SR Adoro system

## Abstract

This report describes a step-by-step sequence for preparation for a posterior partial veneered restoration for a space closure using a Maryland bridge—design veneered with a heat-cured composite resin. The modality provides sound posterior occlusal function, combined with a psychological satisfaction to the patient of regaining a missing tooth. The preparation takes into account the design for resin-bonded prostheses. This article presents the preparation and build-up method for a metal reinforced posterior partial veneered restoration through a conservative palatal approach for a highly aesthetic result.

## INTRODUCTION

The resin-bonded Maryland Bridge has been considered as a popular substitute for conventional full-coverage bridges to avoid crowns on the non-restored abutments. This design has been particularly useful as a conservative option to a single-tooth replacement. Lividitis and Thompson (1980) first developed this technique by electrolytically etching the intaglio surface (inner side) of a non-precious-alloy bridge framework to produce a microscopically roughened surface for providing suitable mechanical retention to the tooth structure through an adhesive luting cement.[1,2] Currently, second-generation designs are based on the same concept of tooth preparation. However, improvements in bonding systems have led to a truly adhesive restoration as opposed to one relying on the micro-etched surface for retention. Adhesive cementation of the alloy to the tooth structure allows the casting to be supported by abutment teeth. The design for the Maryland bridge generally allows for a single path of insertion thus avoiding displacement along any other path except the path of insertion of the prosthesis. Adhesive bonding further strengthens the bond between the framework and the tooth structure, thus increasing the overall success rate of the restoration.

This clinical report describes a conservative method for replacement of a missing posterior tooth using a Maryland bridge with a build-up of heat-cured composite resin (SR Adoro System).

## CASE REPORT

**Figure 1:**
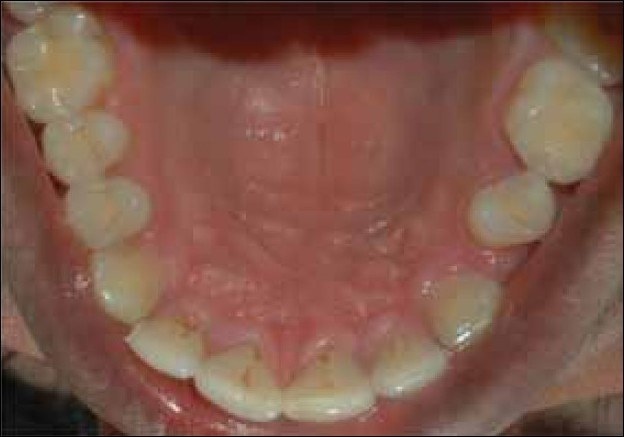
Pre-Operative condition: note rotated maxillary left second premolar and missing first premolar

A 23-year-old male complained primarily of poor aesthetics because of a missing left maxillary premolar. The span of the missing tooth was too small in proportion to the space for the premolar. Both the left maxillary canine and the second premolar were intact. The premolar was rotated approximately 30° clockwise [Figure 1]. The patient was given a wide array of treatment options; including de-rotation of the premolar and a fixed partial denture to a single tooth implant. Since the patient did not agree to spend the time and money required for the treatment, therefore, a resin-bonded fixed partial denture was suggested and he agreed.

**Figure 2:**
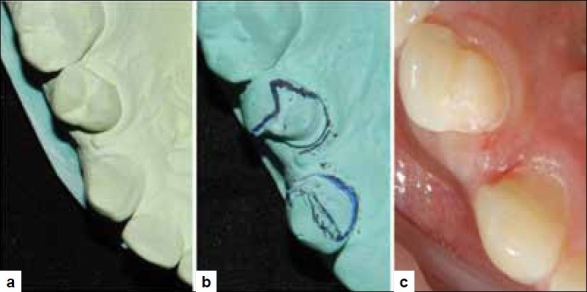
(a) Pre-operative maxillary cast, (b) Mock-up preparation done on duplicated cast to assess tooth reduction and create space for new tooth, (c) Preparation completed on tooth nos 11 and 13

The patient’s occlusion was checked and it was decided that the contact areas would be included within the metal framework of the prosthesis. Accordingly a pre-operative impression was taken in irreversible hydrocolloid (Zelgan, Dentsply, Switzerland) and duplicated. A mock up preparation was done to determine the amount of reduction required on the proximal aspects to compensate for the loss of mesio-distal width [Figure 2a, b].

The clinical preparation was done accordingly by first disking 0.5 mm from either proximal surfaces, i.e. distal surface of the canine and mesial surface of the premolar. A preparation similar to that of a three quarter was done on the premolar and chamfer margin was placed, whereas only the lingual surface reduction was done for the canine[3,4] [Figure 2c].

**Figure 3:**
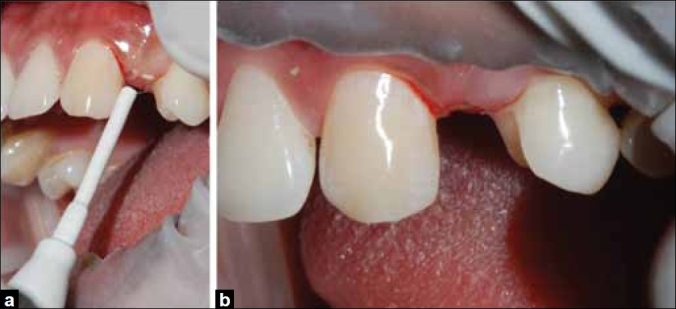
(a) Electrocautery used with loop type attachment to re-contour the interproximal gingiva, (b) Concave surface in the cervical region to provide an emergence profile and maintain crown length

**Figure 4:**
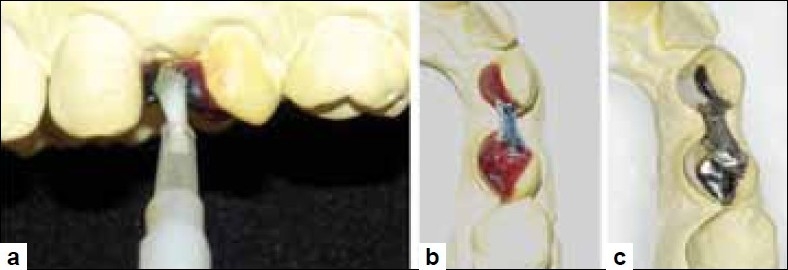
(a) Application of retention adhesive on the fabricated wax pattern, (b) Attachment of micro-retention beads on the connector portion of the wax pattern, (c) Completed metal framework with micro-retention beads seen

After completion of the tooth preparation, the inter-proximal tissue was re-contoured using an electrocautery (Servotome, Satelec, Meriganc, France) with a loop-type attachment to provide an emergence profile for the pontic [Figure 3a, b]. Since there was minimal bleeding, impressions were immediately made using an elastomeric impression material (3M ESPE, ExpressTM STD, USA) and was poured with a high-strength plaster stone (Fujirock, GC Corp.). The wax pattern was fabricated on the master cast and retention beads were placed using a retention adhesive. These were later reproduced in the metal framework [Figure 4].

**Figure 5:**
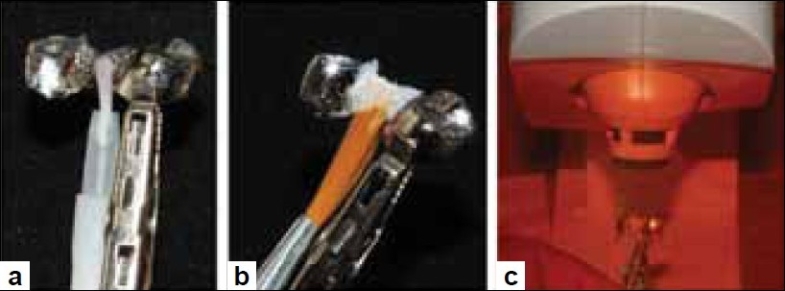
(a) Application of SR link on connector portion of polished metal framework, (b) Opaque layer applied using camel hair brush, (c) Pre-curing each layer for 20 seconds

**Figure 6:**
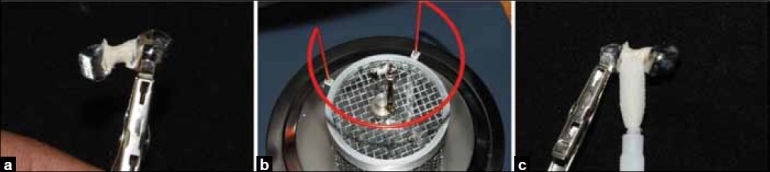
(a) Completed application of opaque layer, (b) Placement in the Lumamat 100 for 15 min., (c) Removal of inhibition layer from opaque coat

**Figure 7:**
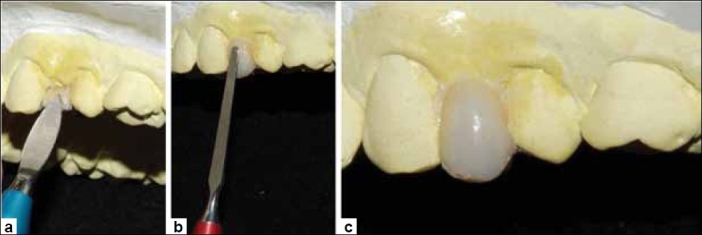
(a) Modelling instrument C used for incremental build-up of the composite, (b) Modelling instrument B used for contouring composite over labial surface, (c) Finished build-up seen from labial surface. Notice the stains and characteristics imparted on the proximo-buccal portions

**Figure 8:**
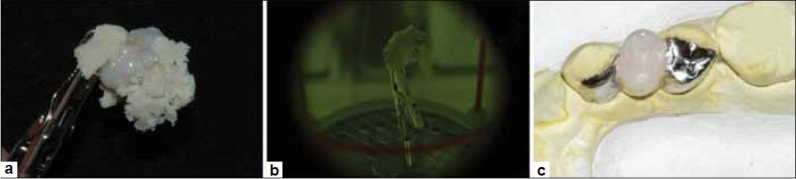
(a) SR Gel placed to cover cured composite build-up and SR adoro thermo guard placed over metallic portion of framework, (b) Metal framework placed in furnace lumamat 100 for curing 25 min. cycle (c) Clean and finished metal supported adoro maryland bridge

The missing tooth was fabricated using the light-activated heat-cured composite resin material (SR Adoro, Ivoclar Vivadent, Germany). The areas of the retention beads were blocked using the Adoro Opaquer material to prevent reflection of the metallic surface [Figure 5]. The opaquer was cured in the curing unit (Lumamat 100, Ivoclar Vivadent, Germany) for 11 min. The inhibition layer was removed using a sponge provided with the system [Figure 6]. A ridge-lap-type pontic of the premolar was designed using incremental addition of composite and layering of stains, while each increment was cured for 20 s under the Quik SET (Ivoclar Vivadent, Germany) [Figure 7]. The completed composite build-up was covered in SR gel placed and SR Adoro Thermo Guard (Ivoclar Vivadent, Germany) placed over metallic portion of framework. The prosthesis was subjected to the final curing cycle for 25 min [Figure 8]. Finished prosthesis was finished using fine and super fine diamond abrasives (Mani Diaburs, Japan) Final polishing was done using polishing discs (SuperSnap, Shofu, Japan).

**Figure 9:**
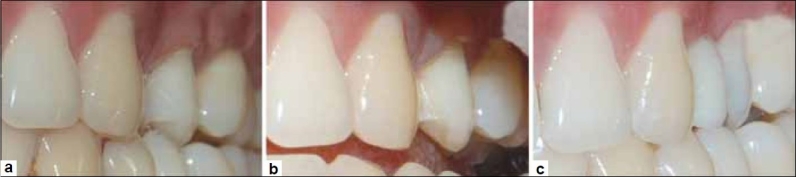
(a) Pre-Operative view, (b) After preparation: Minimal preparation on the mesial surface of second premolar seen from labial aspect, (c) Post cementation: Replaced tooth is in harmony with adjacent teeth in terms of shade and anatomy

**Figure 10:**
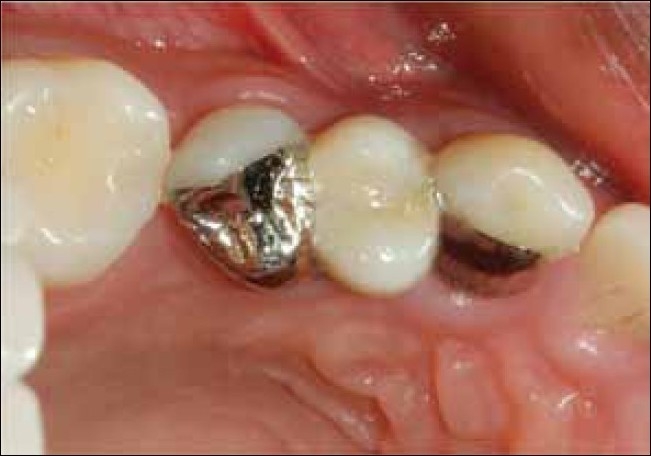
Occlusal view of cemented maryland bridge

At the next visit, the intaglio surfaces of the retainers to be bonded were airborne-particle abraded with 50 *μ*m grain-sized aluminium oxide particles using a grit blaster (Micro Blaster MB102, Comco Inc., Burbank, CA).[5] A metal primer (Multilink System Pack Ivoclar Vivadent, Germany) was applied on the freshly airborne-particle-abraded surface. The bonding surface of the abutments was cleaned with pumice and acid-etched with 37% phosphoric acid, thoroughly washed with water and air-dried. The prosthesis was then seated with an adhesive luting agent (Multilink System Pack Ivoclar Vivadent, Germany). The final prosthesis was in complete anatomical and aesthetic harmony with the adjacent teeth in the dentition [Figures 9 and 10]. The patient followed a regular check-up program after seating of the prosthesis.

## DISCUSSION

This treatment modality presents as a quick and efficient method to provide satisfactory aesthetics with minimal tooth loss. There is less chairside time required and usually no anesthesia is needed. If anesthesia is needed, it is usually for the developing of a soft tissue pontic receiving site on the edentulous ridge or removal of deep carious lesions on the abutment teeth if any. An aesthetic advantage is that only the lingual surfaces of anterior teeth are covered, and therefore no metal from the retainers show.[6] The Adoro System provides an excellent option for build-up of single tooth pontics with a metal supported framework. The ease of use and wide range of shades allow the dentist to fabricate the prosthesis to even the most minute of details to provide a true to natural effect. The composite material can be finished and polished like regular direct composite restoratives.

The only disadvantage to a Maryland Bridge is that it is extremely technique sensitive. Each and every step requires a proper planning and precision including recording impressions, surface treatment of the metal framework, and bonding. Other disadvantages are that it cannot be used for unhealthy or broken down teeth and the metal retainers may show through thin anterior maxillary teeth causing a “graying” problem.[6]

## CONCLUSION

The Maryland Bridge has undergone many alterations since its introduction in 1980, although the basic advantage of conservation of tooth structure has remained. Retention has been improved with a more retentive framework design, the addition of grooves, labial wrap, and the concept of maximum coverage of the enamel. Improvements in material will continue. If the dentist maintains meticulous attention to detail and proper patient selection, the Maryland Bridge will continue to be not only a conservative restoration alternative, but a primary choice. In addition, with all the innovative technology available, variations of the original design are limited only by the imagination of the dentist and the dental laboratory.
